# Two cases of small bowel necrosis due to intussusception secondary to abnormal proliferation of intestinal Peyer’s patches in infants after MMR vaccination

**DOI:** 10.1186/s12887-024-04618-0

**Published:** 2024-02-29

**Authors:** Junzhao Zhu, Weiping Cheng, Youbo Xu, Yingqiang Guo, Lexiang Shi

**Affiliations:** 1 Xi’an International Medical Center Hospital Affiliated to Northwest University, No.777 Xitai Road, Chang’an District, Xi’an City, Shaanxi Province China; 2 The Affiliated Hospital of Northwest University/Xi’an No.3 Hospital, No. 10 East Section of Fengcheng 3rd Road, Weiyang District, Xi’an , China

**Keywords:** Peyer’s patches, Intussusception, Small bowel necrosis, Infants

## Abstract

**Background:**

Intussusception is one of the most common acute abdominal conditions in pediatric patients, and if left untreated, it may result in intestinal necrosis and even death. The etiology of the disease is unknown and may be related to a variety of factors, and there are only limited reports of small bowel necrosis secondary to abnormal Peyer’s node hyperplasia after MMR vaccination.

**Case presentation:**

In this report, we present two infants who had an abnormal proliferation of Peyer’s nodes secondary to intussusception eventually leading to small bowel necrosis after MMR vaccination.

**Conclusions:**

Intestinal necrosis and infectious shock are the most common causes of infant mortality, and early detection and management are critical.

## Introduction

Intussusception is a type of intestinal obstruction that occurs when a segment of the intestine and its corresponding ligament are snapped into the adjacent intestine. There are two types of intussusception: primary and secondary. Almost all infants with intussusception are primary, and the exact cause is unknown, but may be related to dietary changes, their own ileal anatomy, viral infections (vaccination), intestinal autonomic disorders, and genetics. Secondary intussusception is mostly due to organic lesions in the intestinal wall or lumen, with Meckel’s diverticulum and intestinal polyps being the most common.

## Case 1

**Fig. 1:**
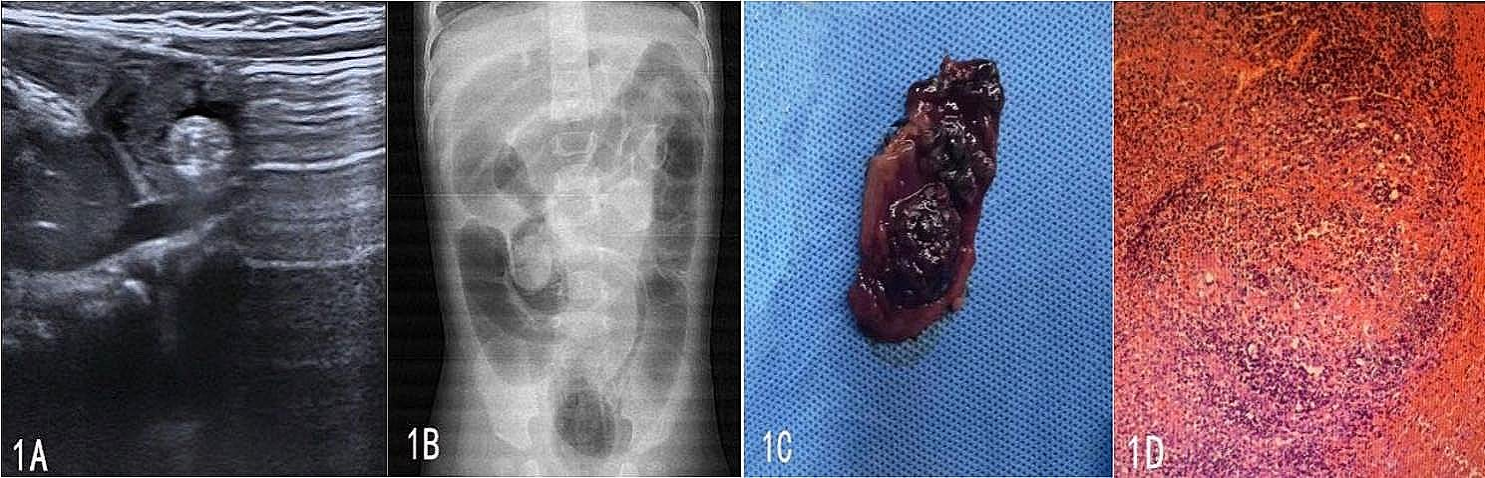
Pictures related to the operation of the case 1. **A** Preoperative ultraphonic results of the child 1. **B** Image of an air enema in progress. **C** The medial wall of the necrotic intestinal canal. **D** Postoperative Pathological results of tumour under the microscope

A 9-month, 15-day-old girl was presented to the emergency department with intermittent vomiting for one day with fever and one episode of blood in the stool. According to her mother, the girl had received the MMR vaccine 5 days earlier and had subsequently been febrile. She was otherwise asymptomatic. On examination, there was distention of the abdomen. A mass was palpable in the right upper abdomen. Laboratory tests performed on admission showed the presence of anemia (RBC 3.77*10^12/L, HGB 102 g/L, HCT 0.32) with a normal white blood cell count and mildly elevated C-reactive protein of 15.2 mg/L (normal value < 3 mg/L). Renal and liver function tests and electrolytes also produced normal results. Blood samples were studied for EB virus and Mycoplasma pneumoniae and subsequently came back negative. Ultrasound (Fig. 1A) showed spherical strong echogenicity in the right intestinal canal of the umbilicus, which could be a foreign body. The chest X-ray did not show any abnormality, and air enema (Fig. 1B) showed a mass-like hyperdense shadow at the hepatic flexure of the colon, and the mass was spherical close to the ileocecal part after repeated pressure, and the reset failed. A diagnostic laparoscopy was performed, and after induction of general anesthesia, the patient was prone. Laparoscopy revealed 300 ml of clarified yellowish exudate in the right iliac fossa, an erythematous appendix with signs of inflammation, complicated intussusception, and a mass approximately 25.0 cm from the ileocecal region that was grayish red in color and approximately 3.0*2.0 cm in size. The whole interstinal canal is 3.0 cm surrounding it was black and necrotic (Fig. 1C). An open bowel resection and appendectomy were performed without any incident. Final histopathological analysis reported abnormal hyperplasia of the intestinal Peyer’s patches, acute appendicitis, and acute suppurative inflammation of the small intestinal mucosa. Postoperative symptomatic treatment was administered, and the patient was discharged on the 12th postoperative day (Fig. 1D).

## Case 2

**Fig. 2:**
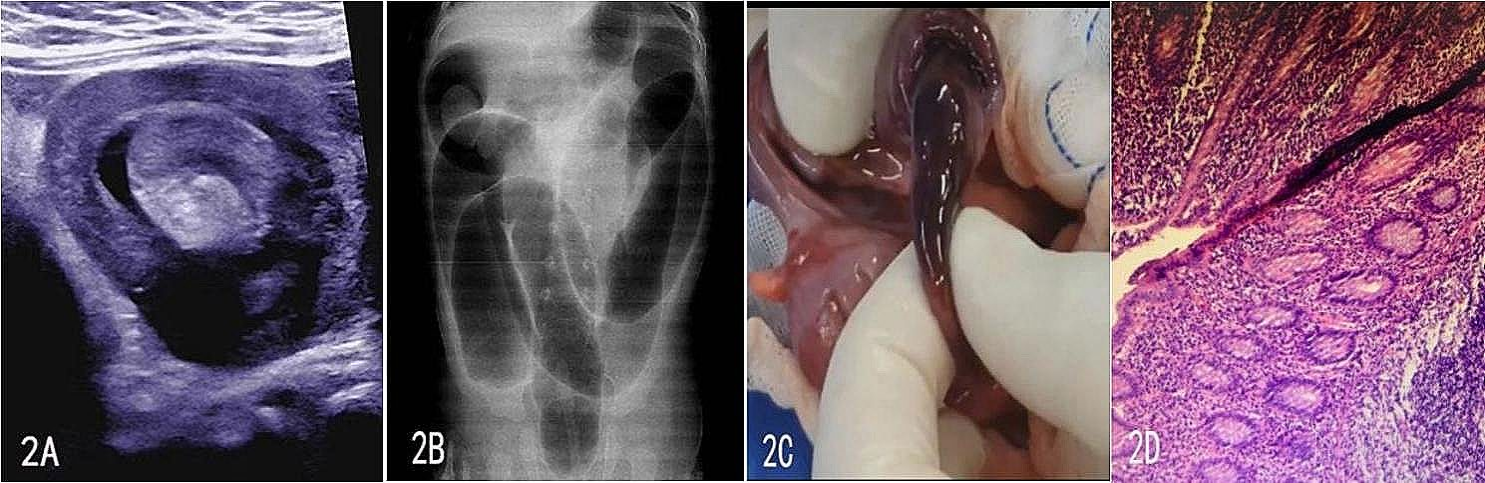
Typical picture of a complex intussusception caused by abnormal proliferation of Peyer’s patches from case 2. 2 **A** Preoperative ultrasound findings of the patient. **B** Image of the air enema being performed. **C** Image of the manipulative repositioning being performed. **D** Microscopic pathological findings of the postoperative lump

A 7-month-old, 23-day-old boy was presented to the emergency department with intermittent vomiting for one day accompanied by fever and two episodes of blood in the stool. According to his mother, the boy had received the MMR vaccine 3 days earlier and subsequently developed a high fever. He was otherwise asymptomatic. On examination, he had a swelling and pain in his abdomen. A lump could be felt on the left side of the umbilicus. Laboratory tests performed on admission showed the presence of anemia (RBC 3.54 * 10^12/L, HGB 87 g/L, HCT 0.28 with a mildly elevated white blood cell count of 9.98 *10^9/L (normal value < 9.5 *10^9/L) and a mildly elevated C-reactive protein level of 13.6 mg/L (normal value < 3 mg/L). Renal and liver function tests and electrolytes were also normal. The blood samples were tested for EB virus and Mycoplasma pneumoniae, and the subsequent results were positive for Mycoplasma pneumoniae but negative for EB virus Ultrasound (Fig. 2A) and showed a mixed echogenic mass in the left lower abdominal intestinal canal, with a range of about 4.0*3.0 cm, exhibiting concentric circles, and a liquid echogenicity in the mixed echogenicity, with a range of about 2.0*1.0 cm. It was probably intussusception with bleeding. The air enema (Fig. 2B ) showed an irregular filling defect in the sigmoid colon. After repeated air injection, the filling defect was seen to shrink and recede to the ascending colon, and the repositioning failed. Diagnostic laparoscopy was performed, and after induction of general anesthesia, the patient was prone. Laparoscopy revealed a right iliac fossa and 300 ml of clarified yellowish exudate, a congested and edematous intestinal canal wall, ileocolic intussusception, and a mass approximately 15.0 cm from the ileocecal region, grayish red in color and approximately 8.0*3.0 cm in size. Its surrounding 14.0 cm intestinal canal was black in color and necrotic. (Fig. 2C). An open enterotomy was performed without any incident. The final histopathological analysis reported abnormal proliferation of intestinal Peyer’s nodes and focal hemorrhagic necrosis of the diseased mucosa. The patient was discharged on the 10th postoperative day after symptomatic treatment (Fig. 2D).

## Discussion

Intussusception is an infant-specific disorder and is the leading cause of gastrointestinal obstruction in infants [1]. The incidence of intussusception in infancy accounts for approximately 60-65% of the overall incidence, with a male-to-female ratio of approximately (2–3):1. Mesenteric lymphoproliferation appears to be the main cause of intussusception in children [2]. Numerous related studies have reported a very strong association between viruses, mesenteric lymphoproliferation and intussusception [3–5]. Geographic and environmental factors play an important part in the incidence of intussusception. The incidence of intussusception varies from country to country; for example, the incidence of pediatric intussusception is higher in China than in the United States [6]. Viral infections are closely associated with intussusception, and most intussusceptions in pediatric patients are idiopathic; in adults, intussusceptions are usually due to intestinal mucosa, intestinal or exogenous conduction sites that act as Pathological lead point regions, pulling the proximal portion of the intestine toward the distal portion for peristalsis [7]. The incidence of intussusception in children under 1 year of age is 0.33/‰ [8]. However, pathological lead points can be found in approximately 25% of patients who undergo surgical treatment [9]. Various infections and pathological factors have been associated with the formation of conduction points, such as enteric adenovirus infection, rotavirus vaccination, intestinal cysts, and lymphomas [10–13]. Recent literature on the subject suggests that the presence of SARS-CoV-2 can act on specific lead point (lymph nodes) and directly cause intussusception [14]. The typical clinical manifestations of intussusception in infants are paroxysmal irritability, vomiting, abdominal masses, and blood in the stool. The clinical presentation was typical in both patients. Abdominal ultrasonography is the standard method for the diagnosis of intussusception. The most common treatment for intussusception is pneumatic or hydraulic enema [15, 16]. When the above-mentioned non-surgical treatment measures are ineffective, surgical treatment must be performed as soon as possible. Delayed surgery can lead to extensive necrosis of the intestine, causing septic shock and leading to multiple organ failure [17]. Surgical treatment is indicated for complex cases with/of small bowel type intussusception, secondary intussusception, and cases in which non-surgical treatment methods have failed. After laparoscopic exploration in both cases, it was clear that it was a complicated intussusception caused by an intestinal bulbous foreign body and some of the intestinal tubes were necrotic, which made it difficult to complete the laparoscopic surgery, so the umbilical incision was prolonged upward for about 4 cm to resect the diseased intestinal tubes, and after resection of the foreign body and necrotic intestinal tubes, normal intestinal tubes were anastomosed, and finally, the abdominal skin wounds were anastomosed.

Infantile intussusception due to abnormal proliferation of intestinal Peyer’s nodes is very rare, and healthcare professionals should be highly vigilant. Possible complications after MMR vaccination, including febrile convulsions, rash, localized lymph node enlargement, and idiopathic purpura, and complications such as mumps (salivary gland involvement) and meningitis/encephalitis (testicular involvement and infection), have also been reported in related cases [18]. This case is the first to report a possible correlation between MMR vaccination and abnormal proliferation of intestinal Peyer’s patches leading to intussusception. The relationship between intussusception and MMR vaccination has not been studied, mainly because there are so few relevant cases. More cases and tests are needed to link the two. The two children in this report had an extremely similar disease course experience, both having received MMR vaccination within 1 week prior to the onset of the disease, with the first manifestations being recurrent high fever and irritability, followed by gradual onset of intermittent vomiting, abdominal distension, and blood-stained stools. Both patients had a complex intussusception(ileal ileocecal intussusception). Based on the authors’ analysis, was the abnormal proliferation of lymphoid tissue in the intestinal Pyle’s nodes that led to intussusception in two infants related to the MMR vaccination prior to the onset of the disease? There may be a relationship, but there is a lack of relevant clinical evidence and more relevant reports and clinical studies are needed. The vaccination reaction masked the early clinical manifestation of intussusception, which led to the delay of the disease, and when the disease became serious at a later stage and manifested itself as intestinal obstruction and necrosis, the optimal treatment period was missed, which seriously jeopardized the growth and development and the life and health of the affected children.

## Conclusion

The etiology of infantile intussusception is unknown, and knowledge of the risk factors for the condition can help its early prevention and treatment. Infantile intussusception due to abnormal proliferation of intestinal Pyle’s nodes is very rare, and pediatricians should be vigilant. Localized lymphoid hyperplasia often occurs after leprosy vaccination, and currently there are too few cases reporting its likely lead to necrosis of intestinal Pyle’s nodes secondary to intussusception, and more cases and data are needed for research. Understanding the possible risk factors for intussusception is essential for the prevention, diagnosis, and treatment of intussusception in infants and for healthy growth in infancy.

## Data Availability

All data generated or analyzed during this study are included in this article and its supplementary information files.

## References

[1] Bines JE, Ivanoff B. Acute intussusception in infants and children: incidence, clinical presentation, and management: a global perspective. World Health Organization; 2002.

[2] Doi O, Aoyama K, Hutson JM (2004). Twenty-one cases of small bowel intussusception: the pathophysiology of idiopathic intussusception and the concept of benign small bowel intussusception. Pediatr Surg Int.

[3] Okimoto S, Hyodo S, Yamamoto M, Nakamura K, Kobayashi M (2011). Association of viral isolates from stool samples with intussusception in children. Int J Infect Dis.

[4] Montgomery EA, Popek EJ (1994). Intussusception, adenovirus, and children: a brief reaffirmation. Hum Pathol.

[5] Konno T, Suzuki H, Kutsuzawa T, Imai A, Katsushima N, Sakamoto M, Adachi M (1978). Human rotavirus infection in infants and young children with intussusception. J Med Virol.

[6] Cai W, Zhang W, Wei G. Pediatric Surgery. 6th Edition. Beijing: People’s Medical Publishing House. 2020.

[7] Cera SM (2008). Intestinal intussusception. Clin Colon Rectal Surg.

[8] Mona Eng P, Mast TC, Loughlin J (2012). Incidence of intussusception among infants in a large commercially insured population in the United States. Pediatr Infect Dis J.

[9] Ntoulia A, Tharakan SJ, Reid JR (2016). Failed intussusception reduction in children: correlation between radiologic, surgical, and pathologic findings. AJR Am J Roentgenol.

[10] Burnett E, Parashar UD, Tate JE (2020). Associations of intussusception with adenovirus, rotavirus, and other pathogens: a review of the literature. Pediatr Infect Dis J.

[11] Tate JE, Yen C, Steiner CA (2016). Intussusception rates before and after the introduction of rotavirus vaccine. Pediatrics.

[12] Lai M, Coakley BA, Webber EM (2018). Intussusception with cecal cyst as lead point in a child. Pediatr Emerg Care.

[13] Bussell HR, Kroiss S, Tharakan SJ (2019). Intussusception in children: lessons learned from intestinal lymphoma as a rare lead-point. Pediatr Surg Int.

[14] Leiva T, Luschen C, Yu Z, Liebe H, Golubkova A, Hunter CJ. COVID-19-Related Intussusception: a Case Series and Review of the literature. Surg Infect (Larchmt). 2022 Aug 18. 10.1089/sur.2022.139. 10.1089/sur.2022.139 35984331

[15] Edwards EA, Pigg N, Courtier J (2017). Intussusception: past, present and future. Pediatr Radiol.

[16] Khan S, Hartman L, Navarro YJS, Rossini CJ, Burdett C, Pennell C (2021). Pediatric Covid-19 mesenteric lymphoid hyperplasia associated intussusception: a case report and literature review. J Pediatr Surg Case Rep.

[17] Cai X, Ma Y, Li S, Chen Y, Rong Z, Li W (2020). Clinical characteristics of 5 COVID-19 cases with non-respiratory symptoms as the first manifestation in children. Front Pediatr.

[18] Abdelbaky AM, Channappa DB, Islam S (2008). Unilateral epididymo-orchitis: a rare complication of MMR vaccine. Ann R Coll Surg Engl.

